# A Performance Comparison between Automated Deep Learning and Dental Professionals in Classification of Dental Implant Systems from Dental Imaging: A Multi-Center Study

**DOI:** 10.3390/diagnostics10110910

**Published:** 2020-11-07

**Authors:** Jae-Hong Lee, Young-Taek Kim, Jong-Bin Lee, Seong-Nyum Jeong

**Affiliations:** 1 Department of Periodontology, Daejeon Dental Hospital, Institute of Wonkwang Dental Research, Wonkwang University College of Dentistry, Daejeon 35233, Korea; seongnyum@wku.ac.kr; 2 Department of Periodontology, Ilsan Hospital, National Health Insurance Service, Goyang 10444, Korea; mailto:youngtaek@nhimc.or.kr; 3 Department of Periodontology, Mokdong Hospital, Ewha Woman’s University School of Medicine, Seoul 07985, Korea; ddsjb333@gmail.com

**Keywords:** artificial intelligence, dental implants, deep learning, supervised machine learning

## Abstract

In this study, the efficacy of the automated deep convolutional neural network (DCNN) was evaluated for the classification of dental implant systems (DISs) and the accuracy of the performance was compared against that of dental professionals using dental radiographic images collected from three dental hospitals. A total of 11,980 panoramic and periapical radiographic images with six different types of DISs were divided into training (*n* = 9584) and testing (*n* = 2396) datasets. To compare the accuracy of the trained automated DCNN with dental professionals (including six board-certified periodontists, eight periodontology residents, and 11 residents not specialized in periodontology), 180 images were randomly selected from the test dataset. The accuracy of the automated DCNN based on the AUC, Youden index, sensitivity, and specificity, were 0.954, 0.808, 0.955, and 0.853, respectively. The automated DCNN outperformed most of the participating dental professionals, including board-certified periodontists, periodontal residents, and residents not specialized in periodontology. The automated DCNN was highly effective in classifying similar shapes of different types of DISs based on dental radiographic images. Further studies are necessary to determine the efficacy and feasibility of applying an automated DCNN in clinical practice.

## 1. Introduction

Dental implants have become a predictable treatment alternative for patients with partial or complete edentulous conditions [1]. Over the years, this treatment modality has evolved as a standard treatment protocol for replacing missing teeth. Thus, hundreds of manufacturers worldwide are producing and distributing over 2000 different types of dental implant systems (DISs) that differ in diameter, length, shape, coating, and surface material and properties [2,3]. Therefore, clinical dental practitioners have to select the appropriate DIS for a specific clinical indication based on their personal skillset and preferences.

DISs have shown a success rate of more than 90% and long-term survival rate of more than 10 years in systematic and meta-analytic review studies, which inevitably increases with the occurrence of mechanical and biological complications, such as fixtures or screw fractures, screw loosening, veneer chipping or fractures, low implant stability, peri-implant mucositis, and peri-implantitis [4,5,6,7]. Therefore, identifying the correct DIS is very important to repair or replace the existing DIS without available information [8,9]. However, studies on methods and techniques that enable the clear identification of DISs are scarce [10,11].

A deep convolutional neural network (DCNN) is a type of artificial intelligence that uses a cascade of multiple layers of nonlinear processing units for feature extraction, transformation, and classification of high-dimensional datasets [12]. A DCNN that is specifically designed for detection, classification, and segmentation in vision tasks and practical applications has been rapidly exploited in recent years in conjunction with improvements in computer performance and deep learning techniques [12]. Particularly, DCNN has been successfully applied in several medical and dental fields, and demonstrated significant advantages in terms of diagnosis and prognosis, such as diabetic retinopathy in retinal fundus photographs, skin cancer in skin lesion photographs, periodontally compromised teeth and dental caries on dental radiographs, and oral cystic lesions on cone beam computed tomography [13,14,15,16,17].

The fine-tuning of deep learning-based algorithms requires specific technical skills and mathematical knowledge, and creating an optimized DCNN for medical and dental applications is an extremely challenging task with numerous hidden challenges [18,19]. Therefore, in recent years, an automated DCNN that regulates the entire deep learning process involved in appropriate model selection and optimized hyper-parameter tuning was developed. The effectiveness and suitability of this automated DCNN are being evaluated in medical applications [20,21].

In the dental field, it is difficult to find studies related to automated DCNN, and to the best of our knowledge, no studies have been conducted on the classification of DISs using fully automated DCNN. We hypothesized that automated DCNN is highly effective in classifying similar shapes of different types of DISs compared to most dental professionals. Therefore, the purpose of this study is to evaluate the efficacy of the automated DCNN for classifying various types of DISs and compare the performance accuracy with dental professionals using dental radiographic images.

## 2. Materials and Methods

### 2.1. Dataset

The study design was approved by the Institutional Review Board of Daejeon Dental Hospital, Wonkwang University (approval no. W2003/003-001). Anonymized raw panoramic and periapical radiographic images (DICOM format panoramic images with a pixel resolution of 2868 × 1504 and periapical images with a pixel resolution of 1440 × 1920) were collected from three multi-center investigations conducted by Daejeon Dental Hospital, Wonkwang University (WKUDH); Ilsan Hospital, National Health Insurance Service (NHIS-IH); and Mokdong Hospital, Ewha Womans University (EWU-MH). The dataset contained six different types of DISs taken between January 2006 and December 2009 at WKUDH and NHIS-IH, and from May 2009 to May 2019 at EWU-MH.

### 2.2. Classification of DISs

DISs were classified into six different types with a diameter of 3.3–5.0 mm and a length of 7–13 mm:

- Astra OsseoSpeed^®^ TX (Dentsply IH AB, Molndal, Sweden), with a diameter of 4.5–5.0 mm and a length of 9–13 mm;
- Implantium^®^ (Dentium, Seoul, Korea), with a diameter of 3.6–5.0 mm and a length of 8–12 mm;
- Superline^®^ (Dentium, Seoul, Korea), with a diameter of 3.6–5.0 mm and a length of 8–12 mm;
- TSIII^®^ (Osstem, Seoul, Korea), with a diameter of 3.5–5.0 mm and a length of 7–13 mm;
- SLActive^®^ BL (Institut Straumann AG, Basel, Switzerland), with a diameter of 3.3–4.8 mm and a length of 8–12 mm;
- SLActive^®^ BLT (Institut Straumann AG, Basel, Switzerland), with a diameter of 3.3–4.8 mm and a length of 8–12 mm.

### 2.3. Data Preparation

Images with severe noise, blur, distortion, and other conditions that impeded the clinical detection and classification of DISs were excluded from the dataset. All included DISs were then manually classified and labeled by five periodontal residents (EHJ, BRN, DHK, JWK, and KYP) who did not directly participate in this study, and confirmed by three participating board-certified periodontists (JHL, YTK, and JBL) based on annotated electronic dental and medical records. A total of 11,980 images, including Astra OsseoSpeed^®^ TX (*n* = 388, 3.2%), Impantium^®^ (*n* = 2512, 21.0%), Superline^®^ (n = 2360, 19.7%), TSIII^®^ (*n* = 5617, 46.9%), SLActive^®^ BL (*n* = 540, 4.5%), and SLActive^®^ BLT (*n* = 563, 4.7%), were extracted from 7146 (59.6%) panoramic and 4834 (40.4%) periapical radiographic images. The details and numbers of radiographic images for each DIS are listed in Table 1. The dataset was randomly divided into two groups: 9584 (80%) radiographic images selected for the training dataset and the remaining 2396 (20%) radiographic images used as the testing dataset. The dataset was resized and transformed into a pixel resolution of 112 × 224, and the brightness and contrast were normalized using the OpenCV library functions [22].

### 2.4. Automated DCNN

**Figure 1:**
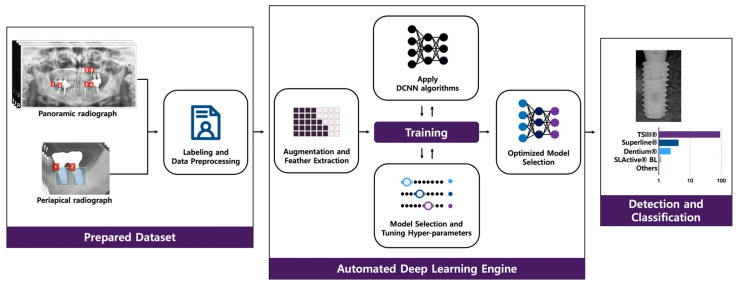
Overview of an automated deep convolutional neural network (DCNN) system.

Automated DCNN using Neuro-T version 2.0.1 (Neurocle Inc., Seoul, Korea), which are specialized tools for automatic model selection and hyper-parameter optimization, were adopted for this study. During training and inference, the automated DCNN automatically creates effective deep learning models and searches the optimal hyperparameters. An Adam optimizer with L2 regularization was used for transfer learning. The batch size was set to 432, and the automated DCNN architecture consisted of 18 layers with no dropout (Figure 1).

### 2.5. Comparing the Performance of the Automated DCNN to that of Dental Professionals

A total of 180 radiographic images (each DIS included 30 panoramic and periapical images) were randomly selected from the test dataset using the Keras framework in Python (version 3.8, Python Software Foundation). We then compared the accuracy of the performance of 25 dental professionals (including six board-certified periodontists, eight periodontology residents, and 11 residents not specialized in periodontology, from WKUDH, NHIS-IH, and EWU-MH) to the trained automated DCNN.

### 2.6. Statistical Analysis

The accuracy of the automated DCNN was evaluated, and the differences between the trained automated DCNN and the dental professionals were compared using the datasets from WKUDH, NHIS-IH, and EWU-MH. For the evaluation, the following statistical parameters were taken into account: receiver operating characteristic (ROC) curve, area under the ROC curve (AUC), 95% confidence intervals (CIs), standard error (SE), Youden index (sensitivity + specificity − 1), sensitivity, and specificity, which were calculated using Neuro-T (version 2.0.1) and R statistical software (version 3.5, R Foundation for Statistical Computing, Vienna, Austria). Delong’s method was used to compare the AUCs generated from the test dataset, and the significance level was set at *p* < 0.05.

## 3. Results

### 3.1. Outcomes of Automated DCNN on the Test Dataset

**Figure 2:**
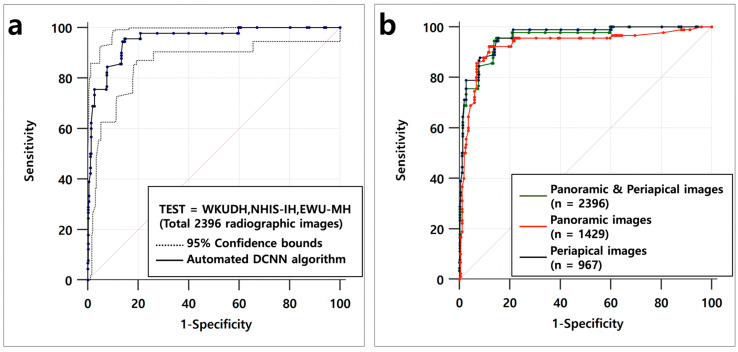
(**a**) Receiver operating characteristic (ROC) curve for classification of six types of DISs in the testing dataset, which consisted of 2396 panoramic and periapical radiographic images. (**b**) The accuracy of the automated DCNN for the test dataset did not show a significant difference among the three ROC curves based on DeLong’s method.

The accuracy of the automated DCNN abased on the AUC, Youden index, sensitivity, and specificity for the 2,396 panoramic and periapical radiographic images were 0.954 (95% CI = 0.933–0.970, SE = 0.011), 0.808, 0.955, and 0.853, respectively. Using only panoramic radiographic images (*n* = 1429), the automated DCNN achieved an AUC of 0.929 (95% CI = 0.904–0.949, SE = 0.018, Youden index = 0804, sensitivity = 0.922, and specificity = 0.882), while the corresponding value using only periapical radiographic images (*n* = 967) achieved an AUC of 0.961 (95% CI = 0.941–0.976, SE = 0.009, Youden index = 0.802, sensitivity = 0.955, and specificity = 0.846). There were no significant differences in accuracy among the three ROC curves (Table 2 and Figure 2).

### 3.2. Outcomes for Automated DCNN Algorithm Compared to that of Dental Professionals

**Figure 3:**
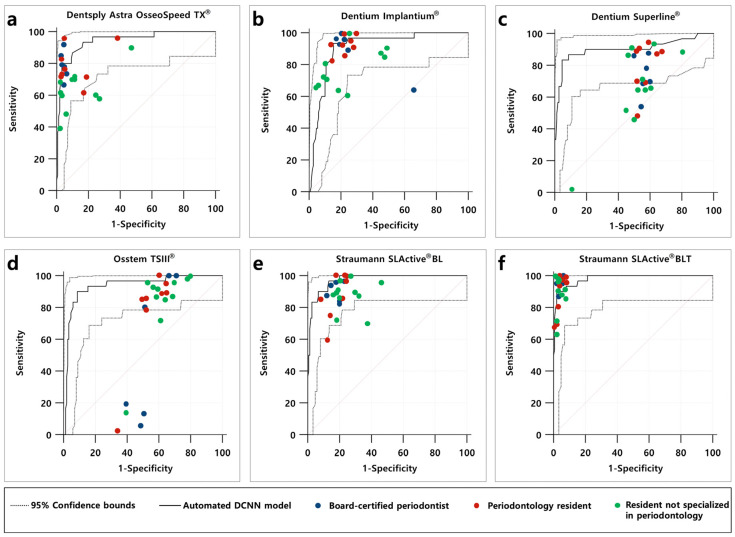
(**a**–**f**) Performance of the automated DCNN and comparison with dental professionals for classification of six types of DISs.

Using 180 panoramic and periapical radiographic images randomly selected from the testing dataset, the automated DCNN outperformed most of the participating dental professionals, including board-certified periodontists, periodontal residents, and residents in other departments, in terms of the overall sensitivity and specificity (Figure 3). In particular, the superior accuracy of the automated DCNN was distinct for Straumann SLActive^®^ BLT (AUC = 0.981, 95% CI = 0.949–0.996, SE = 0.009, Youden index = 0.880, sensitivity = 0.900, and specificity = 0.980) and Straumann SLActive^®^ BL (AUC = 0.974, 95% CI = 0.938–0.992, SE = 0.010, Youden index = 0.833, sensitivity = 0.967, and specificity = 0.867), as shown in Table 3.

## 4. Discussion

Attempts have been made to identify or classify various types of DISs in the past, but most studies have been confined to research in field trials (which use few DIS images or require additional detailed information, such as diameter, length, taper angle, type of thread, and collar shape) [23,24]. Recently, various studies were conducted to confirm the effectiveness of DCNN with respect to identifying various types of DISs [25,26]. As far as we know, this is the first study to use automated DCNN for classifying similar shapes of different types of DISs and demonstrated higher performance accuracy compared to dental professionals.

In our previous studies, we demonstrated that the pre-trained DCNN using dental radiographic images demonstrated high accuracy in identifying and classifying periodontally compromised teeth (AUC = 0.781, 95% CI = 0.650–0.87.6) and dental caries (AUC = 0.845, 95% CI = 0.790–0.901) at a level equivalent to that of experienced dental professionals [15,16]. However, an assessment of clinical parameters (including clinical attachment level, probing depth, bleeding upon probing, tooth mobility, percussion, and electric pulp test), subjective symptoms (including duration and severity of pain and swelling), and radiological interpretation are essential for accurate diagnosis and appropriate treatment. Therefore, the DCNN approach for diagnosing periodontal disease and dental caries using radiographic images has limitations in clinical practice.

In contrast, the DCNN-based approach that uses only radiographic images is very effective and considered to be quite useful in actual clinical practice as a method for classifying various types of DISs with similar diameters and lengths. Two recent studies found that pre-trained or finely tuned DCNN architectures (including VGG16, VGG19, SqueezeNet, GoogLeNet, ResNet-18, MobileNet-v2, and ResNet-50) showed a high accuracy of more than 86% for classifying similar but different types of DISs [25,26]. Our previous study also indicated that the pre-trained DCNN (GoogLeNet Inception-v3) provided reliable results and achieved a higher accuracy (AUC = 0.962, 95% CI = 0.954–0.970) than a board-certified periodontist (AUC = 0.925, 95% CI = 0.913–0.935) for classifying three types of DISs using panoramic and periapical radiographic images [27].

The results of our previous pilot study demonstrated that there is an insignificant difference in the accuracy between panoramic-only and periapical-only based datasets [27]. Moreover, the results of this study confirmed that the accuracy was not statistically or significantly different among the use of panoramic-only (AUC of 0.929, 95% CI = 0.904–0.949), periapical-only (AUC = 0.961, 95% CI = 0.941–0.976), and panoramic and periapical (AUC = 0.954, 95% CI = 0.933–0.970) datasets. Therefore, to compare the accuracy of automated DCNN with that of dental professionals, panoramic and periapical radiographic images were included in one dataset (rather than divided into separate datasets). Additionally, because each DIS used in this study had the same shape but different diameters and lengths, the DISs were not divided according to the diameter and length used to build the dataset.

The Straumann SLActive^®^ BLT implant system has a relatively large tapered shape compared to other types of DISs. Thus, the automated DCNN (AUC = 0.981, 95% CI = 0.949–0.996) and dental professionals (AUC = 0.928, 95% CI = 0.920–0.936) achieved appropriate classifications with high AUC. However, for the Dentium Superline® and Osstem TSIII^®^ implant systems that do not have conspicuous characteristic elements with a tapered shape, the automated DCNN classified correctly with an AUC of 0.903 (95% CI = 0.850–0.967) and 0.937 (95% CI = 0.890–0.967), whereas dental professionals showed a low AUC of 0.541 (95% CI = 0.527– 0.556) and 0.525 (95% CI = 0.510–0.540), respectively. Based on these results, the automated DCNN showed statistically significant higher classification accuracy than dental professionals, including experienced periodontists. Furthermore, it was confirmed that the automated DCNN was highly effective in classifying similar shapes of DISs based on dental radiographic images. Additionally, several previous studies reported that the professional experience of the examiner is an important factor for interpreting dental radiographs [28,29]. Contrastingly, we found that the difference in the experience level associated with DISs did not affect the classification accuracy of DISs significantly because the classification of DISs is unfamiliar regardless of their professional experience.

Nonetheless, this study has certain limitations. Although six types of DISs were selected from three different dental hospitals and categorized as a dataset, the training dataset was still insufficient for clinical practice. Therefore, it is necessary to build a high-quality and large-scale dataset containing different types of DISs. If time and cost are not limited, the automated DCNN can be continuously trained and optimized for improved accuracy. However, owing to computing power constraints, we have to compromise on optimization at the appropriate level. Additionally, the automated DCNN regulates the entire process, including appropriate model selection and optimized hyper-parameter tuning. Therefore, there is less room for human experts to manually check and intervene during the entire process of deep learning training. Cone-beam computed tomography-based three-dimensional images are widely used in the dental field. However, they were not included in the dataset of this study. The classification of DISs using three-dimensional images with less distortion than two-dimensional images is expected to improve accuracy significantly. Therefore, further research is required based on three-dimensional images.

## 5. Conclusions

The selection of an appropriate DCNN model with optimized hyper-parameter tuning is key to the success of deep learning research. We demonstrated that the accuracy of the automated DCNN outperformed most of the participating dental professionals. Therefore, the automated DCNN can help clinical dental practitioners to classify various types of DISs based on dental radiographic images. Nevertheless, further studies are necessary to determine the efficacy and feasibility of applying the automated DCNN in clinical practice.

## Figures and Tables

**Table 1:** Number of panoramic and periapical radiographic images for each dental implant system (DIS).

	Dataset					
	WKUDH		NHIS-IH		EWU-MH	
Dental Implant System	Panoramic images (*n* = 4989)	Periapical images (*n* = 3872)	Panoramic images (*n* = 1120)	Periapical images (*n* = 204)	Panoramic images (*n* = 1037)	Periapical images (*n* = 758)
Dentsply Astra OsseoSpeed TX^®^	247	139	2	-	-	-
Dentium Implantium^®^	589	578	944	148	174	79
Dentium Superline^®^	1011	970	71	32	202	74
Osstem TSIII^®^	2788	1990	100	23	351	365
Straumann SLActive^®^ BL	102	89	3	1	206	139
Straumann SLActive^®^ BLT	252	106	-	-	104	101

Dataset collected from three dental hospitals: Daejeon Dental Hospital, Wonkwang University (WKUDH), Ilsan Hospital, National Health Insurance Service (NHIS-IH), and Mokdong Hospital, Ewha Womans University (EWU-MH). All DISs consist of a diameter of 3.3–5.0 mm and length of 7–13 mm.

**Table 2:** Pairwise comparison of ROC curve for classification of six different types of DISs in the testing dataset.

	Difference between Areas	SE	95% CI	*p*-Value
Panoramic and periapical images vs. oeriapical images	0.007	0.007	−0.008–0.022	0.365
Panoramic and periapical images vs. panoramic images	0.025	0.021	−0.016–0.067	0.235
Panoramic images vs. oeriapical images	0.032	0.020	−0.006–0.072	0.106

AUC, area under the curve; ROC, receiver operating characteristic curve; SE, standard error; CI, confidence interval; AUCs were compared using DeLong’s method for paired ROC curves; panoramic and periapical images, dataset consisting of 2396 panoramic and periapical radiographic images; panoramic images, dataset consisting of 1429 panoramic radiographic images; periapical images, dataset consisting of 967 periapical radiographic images.

**Table 3:** Accuracy comparison between the automated deep convolutional neural network and dental professionals for the classification of six types of DISs, based on 180 panoramic and periapical images randomly selected from the training dataset.

Variables	AUC	95% CI	SE	Youden Index	Sensitivity	Specificity
Dentsply Astra OsseoSpeed TX^®^						
Automated DCNN	0.945	0.901–0.973	0.023	0.766	0.933	0.833
Board-certified periodontists	0.896	0.877–0.914	0.014	0.725	0.777	0.947
Periodontology residents	0.831	0.811–0.850	0.015	0.517	0.570	0.946
Residents not specialized in periodontology	0.777	0.758–0.795	0.014	0.425	0.493	0.931
Dentium Implantium^®^						
Automated DCNN	0.908	0.856–0.946	0.026	0.780	0.933	0.847
Board-certified periodontists	0.791	0.766–0.815	0.013	0.733	0.966	0.766
Periodontology residents	0.806	0.785–0.826	0.011	0.682	0.912	0.770
Residents not specialized in periodontology	0.736	0.716–0.755	0.013	0.465	0.672	0.792
Dentium Superline®						
Automated DCNN	0.903	0.850–0.942	0.041	0.786	0.833	0.954
Board-certified periodontists	0.537	0.507–0.567	0.016	0.333	0.778	0.588
Periodontology residents	0.534	0.508–0.560	0.015	0.330	0.945	0.384
Residents not specialized in periodontology	0.544	0.522–0.566	0.013	0.292	0.884	0.407
Osstem TSIII^®^						
Automated DCNN	0.937	0.890–0.967	0.024	0.813	0.900	0.913
Board-certified periodontists	0.501	0.471–0.532	0.018	0.298	0.911	0.387
Periodontology residents	0.503	0.477–0.529	0.016	0.270	0.104	0.625
Residents not specialized in periodontology	0.556	0.534–0.578	0.014	0.215	0.821	0.394
Straumann SLActive^®^ BL						
Automated DCNN	0.974	0.938–0.992	0.010	0.833	0.967	0.867
Board-certified periodontists	0.759	0.732–0.784	0.015	0.661	0.888	0.772
Periodontology residents	0.753	0.730–0.775	0.014	0.650	0.870	0.779
Residents not specialized in periodontology	0.698	0.677–0.718	0.012	0.507	0.781	0.726
Straumann SLActive^®^ BLT						
Automated DCNN	0.981	0.949–0.996	0.009	0.880	0.900	0.980
Board-certified periodontists	0.968	0.955–0.977	0.011	0.951	0.955	0.995
Periodontology residents	0.915	0.899–0.929	0.014	0.851	0.866	0.985
Residents not specialized in periodontology	0.915	0.902–0.927	0.011	0.852	0.887	0.964
